# Pretreatment Neutrophil to Lymphocyte Ratio Is Associated with Poor Survival in Patients with Stage I-III Non-Small Cell Lung Cancer

**DOI:** 10.1371/journal.pone.0163397

**Published:** 2016-10-03

**Authors:** Jun Wang, Neda Kalhor, Jianhua Hu, Baocheng Wang, Huili Chu, Bicheng Zhang, Yaping Guan, Yun Wu

**Affiliations:** 1 Department of Oncology, General Hospital, Jinan Command of the People’s Liberation Army, Jinan, China; 2 Department of Pathology, The University of Texas MD Anderson Cancer Center, Houston, Texas, United States of America; 3 Department of Biostatistics, The University of Texas MD Anderson Cancer Center, Houston, Texas, United States of America; 4 Department of Oncology, Wuhan General Hospital of Guangzhou Command of the People’s Liberation Army, Wuhan, China; Baylor College of Medicine, UNITED STATES

## Abstract

**Background:**

Neutrophil-to-lymphocyte ratio (NLR) has been shown to be a prognostic indicator in several types of cancer. We aimed to investigate the association between NLR and survival in surgery-treated non-small cell lung cancer (NSCLC) patients.

**Study Design:**

This large retrospective study included 1,245 patients who underwent initial surgery for stage I–III NSCLC at The University of Texas MD Anderson Cancer Center between December 2002 and November 2010. We analyzed the relationship of NLR with clinicopathological variables, local recurrence-free survival (LRFS), distant recurrence-free survival (DRFS), recurrence-free survival (RFS), overall survival (OS), and disease-specific survival (DSS) in patients with high or low NLR using Kaplan-Meier method. Hazard ratios (HRs) with 95% confidence intervals (CIs) were used to assess the prognostic strength of NLR.

**Results:**

There was a statistically significant association between the pretreatment NLR and histology type (*P* = 0.003) and tumor grade (*P* = 0.028). At a median follow-up time of 50.6 months, high NLR was associated with reduced DRFS (*P* = 0.011), OS (*P* < 0.0001) and DSS (*P* = 0.004); it was not associated with LRFS and RFS. Multivariable Cox analysis further revealed that NLR (*P* = 0.027), pathologic stage (*P* < 0.0001) and lymphovascular invasion (*P* < 0.0001) were strong independent predictors for DRFS. NLR was also an independent marker predicting poor OS (*P* = 0.002) and DSS (*P* = 0.017).

**Conclusion:**

The pretreatment NLR can serve as a biomarker to predict distant recurrence and death in stage I–III NSCLC patients. Combination of NLR and pathologic stage can better predict the OS and DSS in stage I-II NSCLC patients.

## Introduction

Non-small cell lung cancer (NSCLC) accounts for approximately 70% to 80% of lung cancers and is the most common cause of cancer-related deaths worldwide. In the past 40 years, progress has been made in the clinical management of NSCLC [1]. Cytotoxic platinum-based chemotherapy has been shown to improve 5-year survival rates when added to surgical resection in patients with stage II–III NSCLC [2]. Chemotherapy also improves the response and prolongs survival in local, advanced, and metastatic diseases [3]. However, fewer than 15% of individuals diagnosed with NSCLC can survive for 5 years. In early-stage NSCLC, only 4% to 5% increases of 5-year survival rates have been achieved, compared with survival prolongation of several months in metastatic diseases [1]. The prognosis of NSCLC patients undergoing surgery is different even for those with the same stage of disease, which suggests there is a heterogeneous population of NSCLC patients with occult metastasis at the time of surgical resection and a risk for relapse.

Currently, the tumor, node, metastasis (TNM) staging system, based on the tumor characteristics, regional lymph nodes, and potential metastatic sites, is a widely accepted prognostic tool for NSCLC patients [3]. Other clinical and pathologic factors used to predict disease progression include patient age at diagnosis and tumor nuclear grade, histologic type, lymphovascular invasion, visceral pleural invasion, and margin status [4,5]. Some molecular markers have been associated with survival [6]. New prognostic factors that can be measured at low cost are needed to enable the identification of specific patients at risk of tumor recurrence and death [7]. These factors are also needed to facilitate the selection of a more aggressive treatment strategy for these patients.

Inflammation and tumor microenvironment is related to cancer development and progression. The specific systematic immune and inflammatory response may be elicited by tissue injury and distortion created by the physical effects of the tumor [8,9]. Systematic inflammatory has also been responsible for cancer-related symptoms including anorexia, pain, debilitation, cachexia, and short survival time [10]. Recent data show that inflammatory cells circulating or accumulating around malignant neoplasms affect tumor progression and patient survival. Some measurable parameters in blood including elevated serum acute-phase proteins, albumin, and increased levels of some cytokines reflect the local and systematic inflammation and lead to the downregulation of immune functions [11]. For example, increased lymphocytic infiltration at diagnosis is associated with an excellent prognosis in cancer patients [12,13]. Patients with high levels of circulating lymphocytes have a better clinical outcome than do patients with low levels of lymphocytes [14] whereas a high density of neutrophils is associated with a poor clinical outcome [15,16]. An elevated level of serum C-reactive protein (CRP) is an indicator of poor prognosis in several cancer types, including lung cancer [17,18]. However, it is not yet established whether any specific component of systemic inflammatory response is better than the other components as a predictor of cancer patient survival.

Recently, high neutrophil-to-lymphocyte ratio (NLR) was shown to worsen outcome in various cancer patients [19–26], including NSCLC and small cell lung cancer [27–33]. However, the significance of the NLR as a predictive marker for recurrence remains unclear in patients with NSCLC. In this large retrospective study, we systematically evaluated the significance of pretreatment NLR and its prognostic function in NSCLC, including local recurrence-free survival (LRFS), distant recurrence-free survival (DRFS), recurrence-free survival (RFS), overall survival (OS), and disease-specific survival (DSS).

## Materials and Methods

### Subjects

Patients were collected from a retrospective cohort of 1,458 patients newly diagnosed with NSCLC at The University of Texas MD Anderson Cancer Center between December 2002 and November 2010. All NSCLC patients were registered from the hospital database. Excluded from our study were NSCLC patients who had previously received neoadjuvant treatment (n = 211), and two patients who did not have data of pretreatment NLR. Thus, a total of 1,245 patients were included in this study. For study-eligible patients, clinicopathologic information including patient sex and age at diagnosis, primary tumor size, pathologic stage, and pretreatment total and differential leukocyte counts (including total white blood cells, neutrophil, monocyte, and lymphocyte counts) from the full blood count routinely performed before surgery were collected for subsequent analysis. The American Joint Committee on Cancer (AJCC) staging system (seventh edition) was used in this retrospective study. NLR was calculated by dividing the absolute neutrophil count by the absolute lymphocyte count. The Cutoff Finder web application tool was used to fit Cox proportional hazard models to dichotomize clinicopathological variables and the survival variables [34]. The optimal cutoff value was defined as the point with the most significant (log-rank test) split [34]. This retrospective study had the approval from Institute Review Board (IRB) at the University of Texas MD Anderson Cancer Center. Each patient was de-identified for the study and the requirement for informed consent was waived by IRB.

### Statistical methods

Data for continuous variables were summarized using the number of subjects, the mean ± standard error (mean ± SE) or median values. Significant differences of clinicopathological parameters between groups were determined with the Mann-Whitney U test, ANOVA test or Kruskal-Wallis test based on the type of the data and comparison. The Spearman rank correlation test was used to analyze the association between NLR and clinicopathological parameters such as tumor size and clinical stage. The primary end points of this study were local LRFS, DRFS, RFS, OS, and DSS rate. LRFS and DRFS durations were defined as the time from the date of diagnosis to the date of locoregional and distant relapse, respectively. The OS time was calculated from the date of diagnosis to the date of death from any cause or to the last date of follow-up. The patient’s OS data were censored at the time of death or at the last follow-up if the patient remained tumor recurrence-free at that time. Survival and follow-up data were obtained from the patient records until August 13, 2013. The univariate analysis of survival differences was carried out with the log-rank test and the Kaplan-Meier method. The Cox proportional hazards model was used to assess the effects of multiple covariates on the survival outcome. Hazard ratios (HRs) estimated from the multivariable analysis were reported as relative risks with the corresponding 95% confidence intervals (CIs). Variables selected from the Cox regression models using the forward stepwise method were also shown to be significantly prognostic in the univariate analysis. All analyses were performed using Prism 6.0 software (GraphPad Software, Inc.) and STATA 11.0 software (Stata Corporation, College Station, TX, USA). For all the analyses, a two-sided *P* value of < 0.05 was considered statistically significant.

## Results

### Patient characteristics

All patient clinicopathological characteristics and therapy modalities are shown in Table 1. The study population comprised 622 men (49.9%) and 623 women (50.1%) patients with NSCLC. The mean age at diagnosis was 65.2 ± 10.3 years, and the median age was 66 years (range, 19–94 years). The tumors were defined as stage I in 775 (62.2%) patients, stage II in 272 (21.9%) patients, and stage III in 198 (15.9%) patients. One hundred seventeen patients had local disease progression, 195 had distant metastases, and 403 patients died of their disease. A total of 295 patients (23.7%) received adjuvant chemotherapy for their NSCLC, and 189 patients (15.2%) received postoperative radiotherapy. The median follow-up time was 50.6 months (range, 0.5–128.6 months). The LRFS, DRFS, RFS, OS, and DSS rates at 5 years were 89.1% (95% CI = 87.0%–91.0%), 82.5% (95% CI = 80.0%–84.6%), 73.4% (95% CI = 70.5%–76.0%), 71.7% (95% CI = 67.8%–73.4%), and 80.6% (95% CI = 77.9%–83.0%), respectively. The median RFS and OS for all patients were 124 and 111 months, respectively, but the median LRFS, DRFS, and DSS have not been reached.

**Table 1:** Clinicopathological features and NLR in NSCLC.

Characteristics	Number of cases (%)	NLR (mean ± SE)	*P*
Age			0.839
≤60	382 (30.7)	3.58 ± 0.24	
>60	863 (69.3)	3.64 ± 0.15	
Sex			0.689
Male	622 (49.9)	3.57 ± 0.20	
Female	623 (50.1)	3.67 ± 0.15	
Race			0.217
White	1116 (89.6)	3.60 ± 0.13	
Black	72 (5.8)	3.71 ± 0.58	
Other	57 (4.6)	3.93 ± 0.52	
Smoking			0.176
Never	241 (19.4)	3.34 ± 0.22	
Previous	711 (57.1)	3.74 ± 0.18	
Current	293 (23.5)	3.57 ± 0.25	
Histology			0.003
SCC	317 (21.3)	3.93 ± 0.24	
AC	722 (60.6)	3.56 ± 0.18	
Other	206 (18.1)	3.37 ± 0.22	
Tumor size (mm)			0.638
≤30	728 (58.5)	3.57 ± 0.17	
>30	517 (41.5)	3.69 ± 0.18	
Node invasion			0.473
Negative	929 (74.6)	3.67 ± 0.15	
Positive	316 (25.4)	3.49 ± 0.21	
Grade			0.028
I	253 (22.3)	3.14 ± 0.16	
II	525 (43.1)	3.74 ± 0.25	
III	467 (34.6)	3.75 ± 0.16	
Surgical procedure			0.285
Lobectomy	1171 (94.7)	3.63 ± 0.13	
Bilobectomy	28 (2.4)	2.89 ± 0.33	
Pneumonectomy	46 (2.9)	3.77 ± 0.44	
LVI			0.830
Negative	1044 (83.9)	3.61 ± 0.14	
Positive	201 (16.1)	3.68 ± 0.28	
VPI			0.070
Negative	928 (74.5)	3.44 ± 0.11	
Positive	317 (25.5)	4.16 ± 0.38	
PNI			0.463
Negative	1220 (98.0)	3.60 ± 0.13	
Positive	25 (2.0)	4.68 ± 1.43	
Adjuvant chemotherapy			0.293
No	950 (76.3)	3.54 ± 0.14	
Yes	295 (23.7)	3.88 ± 0.29	
Adjuvant radiotherapy			0.734
No	1056 (84.8)	3.60 ± 0.13	
Yes	189 (15.2)	3.74 ± 0.39	

SCC, squamous cell carcinoma; AC, adenocarcinoma; TNM, tumor-lymph nodes-metastasis; LN, lymph node; LVI, lymphovascular invasion; VPI: visceral pleural invasion; PNI: perineural invasion

### The correlation of NLR with clinicopathological variables

The mean (mean ± SE) and median NLR was 3.62 ± 0.13 and 2.50 in all NSCLC. As it is shown in Table 1, the NLR was significantly associated with histology type (SCC, 3.93 ± 0.24; AC, 3.56 ± 0.18; *P* = 0.003) and nuclear grade (grade I, 3.14 ± 0.16; grade II, 3.74 ± 0.25; grade III, 3.75 ± 0.16; *P* = 0.028) by Kruskal-Wallis test. A borderline significant association was also found between NLR and visceral pleural invasion (*P* = 0.070). None of the other clinicopathological parameters was significantly related to NLR. Furthermore, a significant correlation was observed between NLR and tumor size by spearman rank correlation test (*r* = 0.07; *P* = 0.019).

### NLR and prognosis in univariate analyses

**Fig 1:**
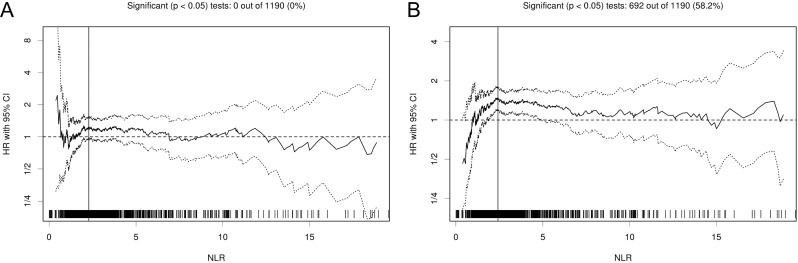
Association of pretreatment NLR with survival in NSCLC. All possible cut-off values for NLR and their impact on RFS (A) or OS (B) are depicted for the whole study cohort. The hazard ratio (HR) including 95% CI is plotted in dependence of the cutoff. A vertical line designates the dichotomization showing the most significant correlation with survival.

**Fig 2:**
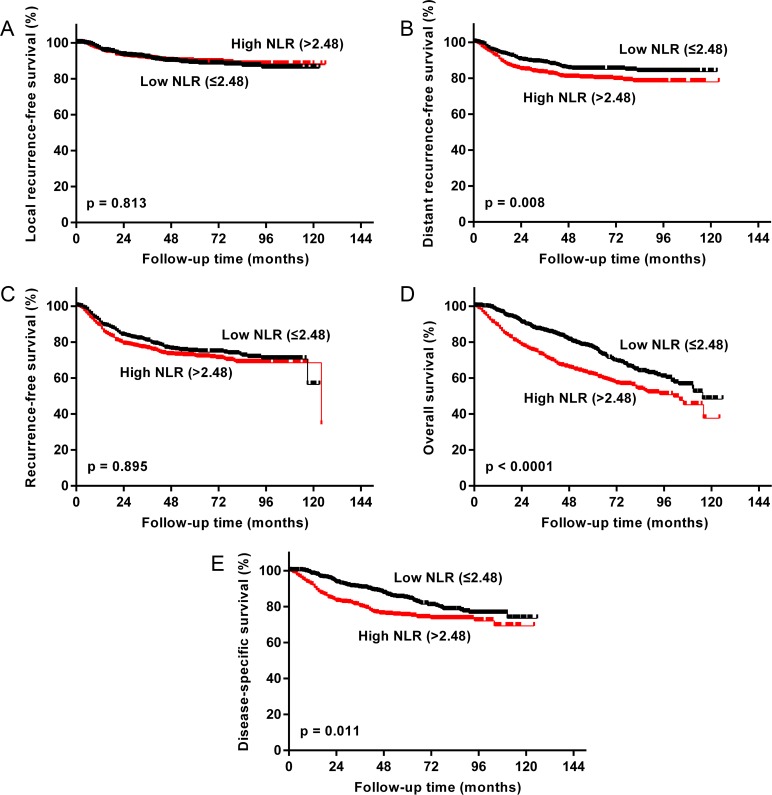
NSCLC survival probabilities in relation to pretreatment NLR. Kaplan-Meier curves for local recurrence-free survival (A), distant recurrence-free survival (B), recurrence-free survival (C), overall survival (D), and disease-specific survival (E) with a 2.48 cutoff in the overall study cohort.

Next, the prognostic cutoff for NLR was set using the Cutoff Finder web application [34]. The statistically optimal cutoff for the separation of a good and poor prognostic NSCLC across all survival parameters (LRFS, DRFS, RFS, OS, and DSS) was at 2.48 (Fig 1). Using univariate analysis, we found a significant difference in prognosis between patients with a high NLR (>2.48) and patients with a low NLR (≤2.48). Patients who had tumors with a NLR ≤2.48 had a significantly longer DRFS (*P* = 0.011), OS (*P* < 0.0001) and DSS (*P* = 0.004), compared with patients who had a higher NLR (Table 2; Fig 2). The DRFS rates for patients with an NLR ≤2.48 and patients with an NLR >2.48 at 5 years were 84.9% (95% CI = 81.5%–87.8%) and 80.1% (95% CI = 76.4%–83.2%), respectively. The RFS rates for patients with an NLR ≤2.48 and patients with an NLR >2.48 at 5 years were 74.7% (95% CI = 70.7%–78.3%) and 72.0% (95% CI = 67.9%–75.6%), respectively. The OS rates for patients with an NLR ≤2.48 and patients with an NLR >2.48 at 5 years were 76.6% (95% CI = 72.6%–80.1%) and 65.1% (95% CI = 60.8%–68.8%), respectively. The DSS rates for patients with an NLR ≤2.48 and patients with an NLR >2.48 at 5 years were 84.4% (95% CI = 80.7%–87.4%) and 76.9% (95% CI = 72.9%–80.4%), respectively.

**Table 2:** Univariate survival analysis (Kaplan-Meier) on LRFS, DRFS, RFS, OS and DSS in selected subgroups of patients according to characteristics.

		LRFS			DRFS			RFS			OS			DSS	
Characteristics	HR	95% CI	*P*	HR	95% CI	P	HR	95% CI	P	HR	95%CI	P	HR	95%CI	P
NLR															
Low	1			1			1			1			1		
High	0.96	0.07–1.38	0.813	1.44	1.08–1.92	0.011	1.00	0.98–1.03	0.895	1.44	1.18–1.76	<0.0001	1.47	1.14–1.91	0.004
Age (years)															
≤60	1			1			1			1			1		
>60	1.12	0.75–1.68	0.572	0.83	0.61–1.11	0.202	0.98	0.72–1.17	0.491	1.33	1.06–1.66	0.013	1.16	0.87–1.55	0.299
Sex															
Female	1			1			1			1			1		
Male	1.47	1.02–2.12	0.041	1.45	1.09–1.93	0.009	1.44	1.15–1.81	0.002	1.46	1.20–1.78	<0.0001	1.78	1.37–2.32	<0.0001
Race															
White	1			1			1			1			1		
Black	1.00	0.44–2.28	0.997	0.87	0.45–1.70	0.69	0.94	0.56–1.57	0.804	1.41	0.95–2.09	0.085	1.16	0.66–2.02	0.613
Other	0.99	0.41–2.45	0.998	0.97	0.48–1.98	0.94	1.02	0.59–1.78	0.942	0.98	0.60–1.62	0.948	1.05	0.56–1.97	0.888
Smoking															
Never	1			1			1			1			1		
Previous	1.71	1.00–2.93	0.051	1.27	0.86–1.87	0.239	1.35	0.99–1.85	0.060	2.21	1.59–3.08	<0.0001	1.97	1.30–2.99	0.001
Current	1.32	0.71–2.48	0.379	1.24	0.79–1.95	0.339		0.79–1.66	0.460	2.18	1.52–3.12	<0.0001	2.03	1.29–3.22	0.002
Histology															
SCC	1			1			1			1			1		
AC	0.79	0.53–1.18	0.252	1.23	0.87–1.73	0.246	1.04	0.80–1.36	0.740	0.79	0.64–0.98	0.036	0.84	0.63–1.12	0.242
Other	0.58	0.31–1.07	0.079	1.15	0.73–1.82	0.544	0.83	0.57–1.20	0.314	0.73	0.53–0.99	0.044	0.82	0.55–1.22	0.322
TNM stage															
I	1			1			1			1			1		
II	2.67	1.76–4.07	<0.0001	3.04	2.17–4.26	<0.0001	2.89	2.21–3.77	<0.0001	2.03	1.60–2.56	<0.0001	2.78	2.04–3.77	<0.0001
III	2.95	1.96–4.68	<0.0001	4.23	2.99–5.97	<0.0001	3.65	2.76–4.84	<0.0001	2.63	2.06–3.37	<0.0001	3.99	2.92–5.46	<0.0001
LN															
Negative	1			1			1			1			1		
Positive	1.06	0.98–1.15	0.166	1.12	1.08–1.17	<0.0001	1.11	1.08–1.15	<0.0001	1.14	1.11–1.18	<0.0001	1.16	1.12–1.19	<0.0001
Tumor size (mm)															
≤30	1			1			1			1			1		
>30	1.82	1.26–2.61	0.001	1.91	1.44–2.53	<0.0001	1.95	1.55–2.44	<0.0001	1.49	1.22–1.81	<0.0001	1.59	1.23–2.05	<0.0001
Grade															
I	1			1			1			1			1		
II	1.51	0.88–2.57	0.133	2.22	1.35–3.65	0.002	1.91	1.32–2.76	0.001	2.51	1.77–3.56	<0.0001	2.81	1.72–4.58	<0.0001
III	1.53	0.88–2.64	0.130	3.06	1.87–5.02	<0.0001	2.31	1.60–3.34	<0.0001	2.99	2.11–4.24	<0.0001	3.66	2.29–5.95	<0.0001
LVI															
Negative	1			1			1			1			1		
Positive	1.13	0.69–1.85	0.616	2.47	1.81–3.37	<0.0001	1.94	1.49–2.52	<0.0001	1.53	1.20–1.95	0.001	1.67	1.22–2.28	0.001
VPI															
Negative	1			1			1			1			1		
Positive	1.52	1.03–2.58	0.033	1.44	1.06–1.95	0.02	1.53	1.19–1.95	0.001	1.35	1.08–1.68	<0.0001	1.55	1.17–2.04	0.002
PNI															
Negative	1			1			1			1			1		
Positive	3.76	1.65–8.56	0.002	2.23	0.99–5.02	0.054	2.61	1.39–4.92	0.003	2.64	1.54–4.49	<0.0001	3.92	2.19–7.02	<0.0001

LRFS: local recurrence-free survival; DRFS, distant recurrence-free survival; RFS: recurrence-free survival; OS, overall survival; DSS, disease-specific survival; NLR, neutrophil to lymphocyte ratio; *n*, number of cases; HR, hazard ratio; NS, not significant; CI, confidence interval; TNM, tumor-lymph nodes-metastasis; SCC, squamous cell carcinoma; AC, adenocarcinoma; LN, lymph node; LVI, lymphovascular invasion; VPI: visceral pleural invasion; PNI: perineural invasion

### NLR and prognosis in multivariable analyses

Multivariable analyses were performed with the Cox proportional hazards model. Patient sex and age, tumor histology, grade, pathologic stage, histologic type, NLR, lymphovascular invasion, smoking status, pleural invasion, visceral pleural invasion and grade were selectively included in the different models for adjustment, and factors that had prognostic significance in the univariate analysis, *P* < 0.05, were included. In that model, we demonstrated that patients with a higher pretreatment NLR were 1.38 times as likely to develop distant relapse as compared to those with a lower pretreatment NLR (95%CI: 1.04–1.84; *P* = 0.027; Table 3). Multivariable analyses also showed that patients with higher NLR had significantly poorer prognoses for both OS (HR: 1.37; 95%CI: 1.12–1.67; *P* = 0.002) and DSS (HR: 1.37; 95%CI: 1.06–1.78; *P* = 0.017) than did patients with lower NLR (Table 3).

**Table 3:** Multivariable analysis of independent prognostic factors for survival in patients with stage I-III NSCLC.

Group	LRFS		DRFS		RFS		OS		DSS	
	HR (95% CI)	*P*	HR (95% CI)	*P*	HR (95% CI)	*P*	HR (95% CI)	*P*	HR (95% CI)	*P*
Age, years (>60 *vs* ≤60)							1.29 (1.02–1.62)	0.030		
Sex (male vs female)	1.37 (0.94–1.99)	0.098	1.21 (0.91–1.62)	0.191	1.28 (1.02–1.61)	0.036	1.21 (0.99–1.48)	0.069	1.52 (1.16–1.99)	0.003
Stage (II, III *vs* I)	2.53 (1.73–3.69)	<0.0001	2.97 (2.20–4.02)	<0.0001	2.78 (2.19–3.54)	<0.0001	2.02 (1.65–2.48)	<0.0001	2.72 (2.07–3.57)	<0.0001
Grade (III *vs* I, II)	0.95 (0.79–1.15)	0.618	1.5 (1.00–1.33)	0.060	1.07 (0.95–1.20)	0.242	1.04 (1.00–1.22)	0.055	1.11 (0.97–1.27)	0.120
Pathology (Other *vs* SCC)							0.97 (0.78–1.20)	0.751		
NLR (High vs low)			1.38 (1.04–1.84)	0.027			1.37 (1.12–1.67)	0.002	1.37 (1.06–1.78)	0.017
LVI (positive *vs* negative)			1.89 (1.37–2.60)	<0.0001	1.44 (1.10–1.90)	0.008	1.18 (0.91–1.52)	0.213	1.16 (0.83–1.60)	0.384
Smoking (Yes *vs* No)							1.92 (1.38–2.68)	<0.0001	1.63 (1.07–2.46)	0.021
VPI (positive *vs* negative)	1.28 (0.86–1.90)	0.231	1.03 (0.75–1.41)	0.869	1.17 (0.91–1.51)	0.219	1.16 (0.93–1.45)	0.199	1.24 (0.93–1.64)	0.148
PNI (positive *vs* negative)	2.73 (1.17–6.34)	0.020			1.42 (0.75–2.71)	0.286	2.1 (1.22–3.67)	0.008	2.32 (1.26–4.29)	0.007

LRFS: local recurrence-free survival; DRFS, distant recurrence-free survival; RFS: recurrence-free survival; OS, overall survival; DSS, disease-specific survival; HR, hazard ratio; CI, confidence interval; LN, lymph node; NLR, neutrophil to lymphocyte ratio; LVI, lymphovascular invasion; VPI: visceral pleural invasion; PNI: perineural invasion

**Fig 3:**
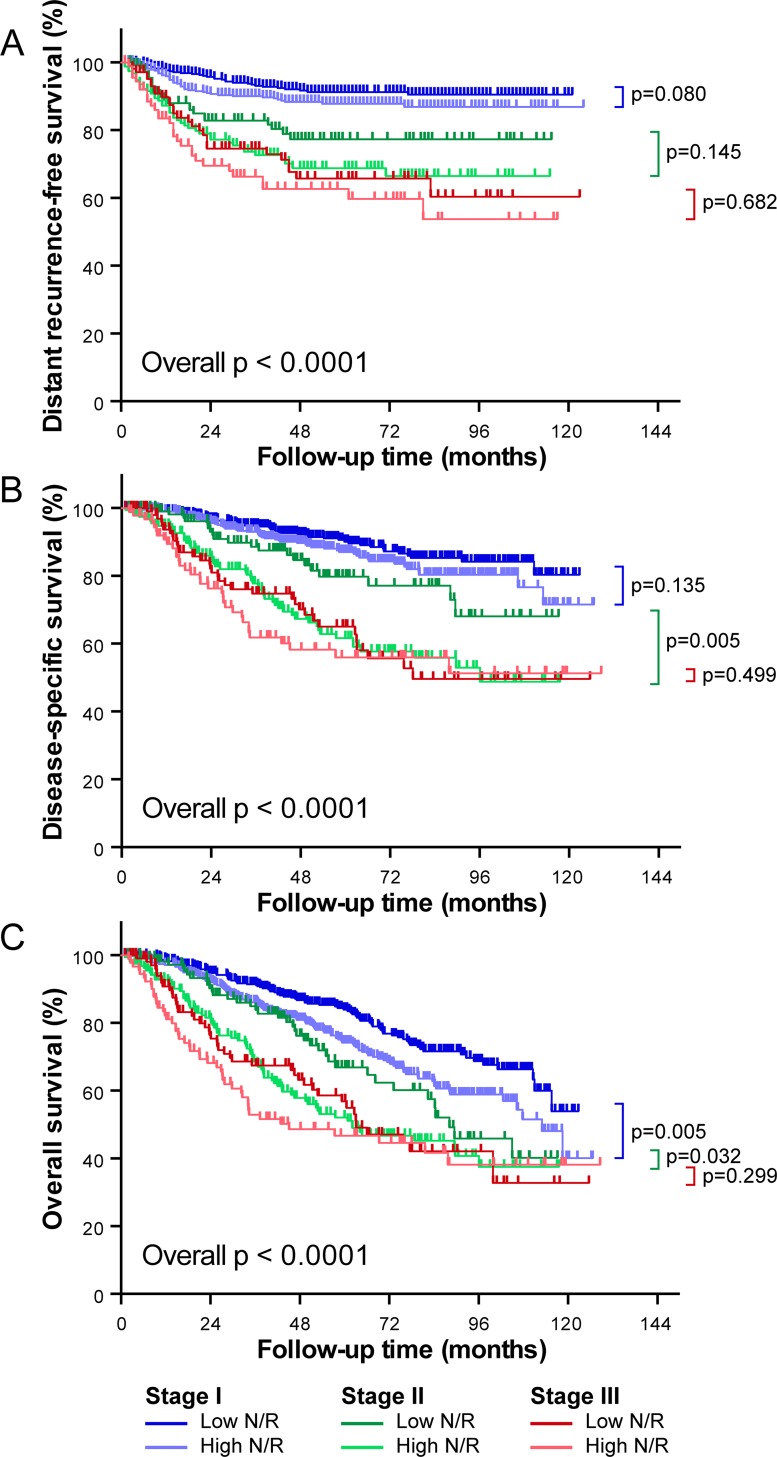
Kaplan-Meier survival curves for NSCLC patients in relation to pathologic stage and NLR. *P* values were determined by the log-rank test. (A) Distant recurrence-free survival, (B) disease-specific survival, and (C) overall survival. Patients with high NLR had shorter OS compared to those with low NLR with stage I disease (*P* = 0.005). Patients with high NLR had both DSS and OS shorter than those in patients with low NLR in stage II disease (*P* = 0.005 and 0.032, respectively).

Since NLR and pathologic stage were independent predictors for clinical outcomes (Table 3), we next combined these two independent variables to determine whether they could better predict survival. As shown in Fig 3, NLR added additional prognostic value to pathologic stage to predict DSS and OS, particular in stage I and II NSCLC patients. Patients with high NLR had shorter OS compared to those with low NLR with stage I disease (*P* = 0.005). Patients with high NLR had both DSS and OS shorter than patients with low NLR with stage II disease (*P* = 0.005 and 0.032, respectively).

## Discussion

To the best of our knowledge, the current retrospective study is one of the largest to analyze the prognostic value of preoperative NLR in the peripheral blood of patients with NSCLC in a single institute. Our study showed that high preoperative NLR is a significant independent predictor of DRFS, OS, and DSS in NSCLC, whereas pretreatment NLR is not associated with overall RFS.

Some reports have showed that NLR could be used as a predictor of poor outcome and treatment response; however, there is no precisely defined cutoff value of NLR yet. The cutoff value of NLR differs among previous studies. In some studies, the patients were separated into two groups according to median value of NLR [20,35]. Other studies dichotomized data directly and used an arbitrary cutoff point of >5 to define a higher NLR in keeping with the previously published literature [36]. The maximum point of sensitivity plus specificity could be used as the optimal cutoff value of NLR for survival, according to ROC analysis. The cutoff values calculated from ROC analysis seem to be suitable to evaluate the effect of NLR on survival [37,38]. Here, we continuously plotted HRs for all possible cut-offs for LRFS, DRFS, RFS, OS and DSS using an automated web software tool [34]. High NLR was significantly associated with a worse prognosis almost over the whole range of potential cut-off values for DRFS, OS and DSS. The statistically optimal cutoff of NLR for the separation of a good and a bad prognostic NSCLC across all survival parameters was at 2.48. However, further standardization for choosing optimal cutoff values of NLR in different tumor types and different stages is needed.

Some studies have pointed out a significant association between pretreatment NLR and poor survival in a variety of cancers, such as colon [25], renal [21], ovarian [26], pancreatic [22], gastric [24], and oral cavity squamous cell cancers [20]. Our results are consistent with previous observations in terms of the association between NLR and survival in lung cancer. Several studies evaluated the association between NLR and outcome in patients with early-stage NSCLC. Sarraf *et al* found that elevated preoperative NLR was associated with higher tumor stage. However, elevated preoperative NLR remains an independent predictor of overall survival rate after complete resection of primary lung cancer and is a potential biomarker to stratify high risk of death in patients with stage I disease [39]. In a retrospective study of 284 NSCLC patients, Tomita *et al* found that the 5-year overall survival rate of patients with high NLR was significantly shorter than that of patients with low NLR [40]. Pinato *et al* analyzed the prognostic performance of inflammation-based prognostic indices in 220 patients with primary operable NSCLCs. The NLR could be used as a predictor of overall survival, with tumor stage and NLR being confirmed as independent prognostic factors on multivariable analyses. However, neither NLR nor other markers predicted shorter time to recurrence after surgery [41]. In the present study, NLR was a significant predictor of distant relapse in NSCLC.

As an inflammatory-immunological marker, the pretreatment NLR was evaluated as an indicator for prognosis in late-stage NSCLC patients [27,42–44]. This baseline NLR is also a useful prognostic predictor for specific populations, such as elderly patients [44], patients receiving first-line gefitinib [42], and patients receiving stereotactic radiation therapy [43]. The variation of NLR during the first cycle of treatment may indicate survival improvement in patients with a poor prognosis [27]. In addition, the combined use of NLR and other inflammatory-immunological markers has been found to have potential prognostic value in patients with NSCLC. For example, the combination of NLR and CRP or the platelet-to-lymphocyte ratio (PLR) and the inflammation index at diagnosis were developed to assess prognosis [41]. Using inflammation index (ALI) at diagnosis as an inflammation marker, Jafri *et al* found that advanced lung cancer patients who have an ALI score of < 18 were significantly more likely to have more than 2 sites of metastatic disease, to have poor performance status and less likely to receive any chemotherapy [45]. Inflammation-based scoring systems, including the Glasgow Prognostic Score (GPS) determined by serum levels of CRP, NLR and PLR, are novel predictors of outcome in cancer patients, and have been extensively used in a variety of clinical scenarios, such as operable patients, chemo/radiotherapy, and inoperable patients [44,46,47]. Furthermore, the NLR at baseline can predict bevacizumab benefit in advanced NSCLC patients [48]. Go *et al* retrospectively analyzed the clinical characteristics, laboratory parameters, and NLR in 114 lung cancer patients newly diagnosed with venous thromboembolism (VTE) and found that the NLR at the time of VTE diagnosis was statistically correlated with the patients’ poor response to anti-coagulation [49]. A recently published study indicated that NLR and platelet to lymphocyte ratio before treatment can be useful biomarkers to assist the diagnosis of lung cancer [23]. In that study, the NLR value was significantly higher in lung cancer patients than in healthy subjects (4.42 vs 2.45). These findings will support the potential role of NLR in the clinical management of NSCLC patients.

The biology of elevated NLR remains unclear. It has been widely accepted that tumor development is associated with inflammation and immunity. Tumor progress and spread are not only related to the intrinsic characteristics of cancer cells but also to the cancer microenvironment. Inflammatory cells including leukocytes and lymphocytes play an important role in controlling proliferation, survival, and migration of tumor cells through apoptosis and angiogenesis pathways. Many studies investigated tumor-associated inflammatory cells’ functions in cancer progression and metastasis. For example, tumor-associated neutrophils can have an anti-tumorigenic phenotype and a pro-tumorigenic phenotype capable of supporting tumor growth and suppressing the anti-tumor immune response [50]. Co-culture of neutrophils and lymphocytes from healthy populations leaded to the suppression of the cytolytic activity of different immune cells including lymphocytes, activated T cells, and natural killer cells [51,52]. Alternatively activated tumor-associated macrophages are relatively poor at killing intracellular pathogens and are involved in tumor growth, angiogenesis, lymphangiogenesis, and immunosuppression [53]. These inflammatory cells can promote tumor growth and metastasis by remodeling the extracellular matrix and releasing indicators to inhibit the function of cytotoxic lymphocytes and change the biological features of tumor cells. Commonly, the systematic inflammatory response causes neutrophilia and relative lymphocytopenia. Enhanced NLR, reduced performance status, and increased C-reactive protein levels could be indicators of reduced immunity functions in individual patients.

Some limitations exist in our study. This retrospective study was conducted in one single institution although with a large number of included cases. Some potential co-factors related to systematic inflammation and immunity have not been considered in all analyses. However, as a marker of inflammation and immunology, NLR is highly repeatable, inexpensive, and widely available.

In conclusion, increasing pretreatment NLR was associated with decreased survival rate after adjustment for known prognostic factors such as clinical stage. Elevated pretreatment NLR might be a potential biomarker predicting relapse and mortality for advanced NSCLC patients. Larger, prospective, and randomized studies are needed to confirm these findings and to elucidate the potential mechanism of systematic inflammatory response against tumor cells.
